# Supplementary material for: Resource landscape shapes the composition and stability of the human vaginal microbiota
Source: PLoS Biol. 2026 Feb 3;24(2):e3003575. doi: 10.1371/journal.pbio.3003575 (PMC12867229; doi:10.1371/journal.pbio.3003575)
Supplement: Attachment — Submitted filename: response_PLOS_BIOL_R1.pdf [file pbio.3003575.s002.pdf]

Responses to the senior editor:

---

a) Please address the comments from the Academic Editor (see the foot of this email).

Reply: Please find our responses to the Academic Editor below.

---

b) We thought that your paper would be better considered as a Short Report. I've changed the article type accordingly, but there is no need for further re-formatting.

Reply: We are happy with this decision. Bearing this in mind, we tried to shorten some formulations.

---

c) Many thanks for providing the necessary data and code. Please cite its location clearly in all relevant main and supplementary Figure legends (Figs 2ABCD, 3AB, S1, S2, S3, S4, S5), e.g. "The data and code needed to generate this Figure can be found at https://doi.org/10.57745/3GJF2Z"

Reply: We have incorporated the suggested sentence throughout the manuscript.

---

d) Your Financial Disclosure statement says "The author(s) received no specific funding for this work." However, in your Acknowledgements, you say "The authors acknowledge support from the Fondation pour la Recherche Medicale (SPF202005011951to TK), the Centre National de la Recherche Scientifique, the Agence Nationale de la Recherche contre le Sida (ANRS—MIE, to NT), the US National Institute of Health (NR015495 to JR)." Please transfer this information into the metadata on the submission form.

Reply: We updated the financial disclosure, especially with the necessity to find support for the publication fee.

---

e) Please include the following wording in your Acknowledgements, in order to recognise the contribution of PCI: "Version 4 of this preprint has been peer-reviewed and recommended by Peer Community In Ecology (https://doi.org/10.24072/pci.ecology.100777)."

Reply: Thank you for helping highlight this open peer-review process!

---

f) Please ensure that the references in your supplement are also present in your main references, to ensure that due credit is received.

Reply: Thank you for pointing this out. We have now included the references cited in the Supporting Information in the bibliography in the main text.

A Response to the academic editor:

As a non-expert, I was wondering if there would also be a possibility to reduce the private resource in real interventions rather than providing more of the shared resource(s). I may have missed it, but this possibility does not seem to be discussed.

Reply: Thank you for this suggestion. Targeting the private goods indeed appears as a promising idea. We now discuss several possibilities that may aid in reducing the private resource (L264-283).

I found the paragraph starting from l. 176 a bit awkwardly phrased. From a mathematical standpoint, it is obvious that once one consumer is lost, it cannot reappear except via "seeding". I think that the authors want to highlight

that the bistability leads to different amounts of seeding that are necessary to bring the system into the basin of attraction of the other stable state, but the phrasing made me think of a trivial statement at first.

Reply: Thank you for pointing this out. We now clarify the problem in terms of bistability and initial conditions.

A major appeal of this paper is that the authors used data to evaluate their predictions by developing ways to break down the real-life systems into simple parameters. However, I was wondering why they decided to use the dominant group as a metric in figure 3, rather than focusing on the proportions of CSTIV like in the model predictions shown in figure 2. Moreover, I would be very curious to hear whether the authors found signals of their predictions in individual time series when the community shifted in the same woman over time.

Reply: We have made two changes following these suggestions from the Academic Editor:

First, we now provide a new supplementary figure (Figure S3 below) demonstrating the frequency of key bacterial species as a function of χ (the relative supply of private resource). We hope to emphasise here that this alternative visualisation demonstrates qualitatively identical patterns seen in Figure 3 in the main text. This is because CSTs and species frequencies have high concordance: CSTs I, II, III and V are dominated by a single *Lactobacillus* species, though CST IV-associated bacteria are more diverse. We chose to keep the original figure in the main text, as we believe it allows for a single-panel representation without a loss of information, but this is, of course, open to discussion.

Second, following the question regarding transitions within individuals, we have carried out an additional statistical analysis (using linear mixed-effects models) and incorporated an additional figure (new Figure 4). These new additions demonstrate that transitions between vaginal microbiota communities within individuals are associated with changes in the proportion of supplied resource private to CST IV, χ. Please find the new paragraph elaborating this result at L241. A small paragraph was added in the Method section to describe the analysis (L508-L513).

B Response to the technical requests:

---

1) We ask that a manuscript source file is provided at Revision. Please upload your manuscript file as a .doc, .docx, .rtf or .tex.

Reply: We now upload a $\TeX$ file.

---

2) Please provide separate figure files in .tif or .eps format. For more information about figure files please see our guidelines: https://journals.plos.org/plosbiology/s/figures

Reply: We now use an EPS format.

---

3) We noticed that you used "unpublished data" in the manuscript. We do not allow these references, as the PLOS data access policy requires that all data be either published with the manuscript or made available in a publicly accessible database. Please amend the supplementary material to include the referenced data or remove the references.

Reply: While we mention the phrase "previously unpublished data" (meaning that the data shown is original), please note that the data are made available online at `https://doi.org/10.57745/3GJF2Z` as now indicated throughout the manuscript. Please let us know if further actions are required.

---

4) We have noticed that you have uploaded Supporting Information files, but you have not included a list of legends. Please add a full list of legends for your Supporting Information files after the references list.

Reply: We have included a list of Supporting Information legends.

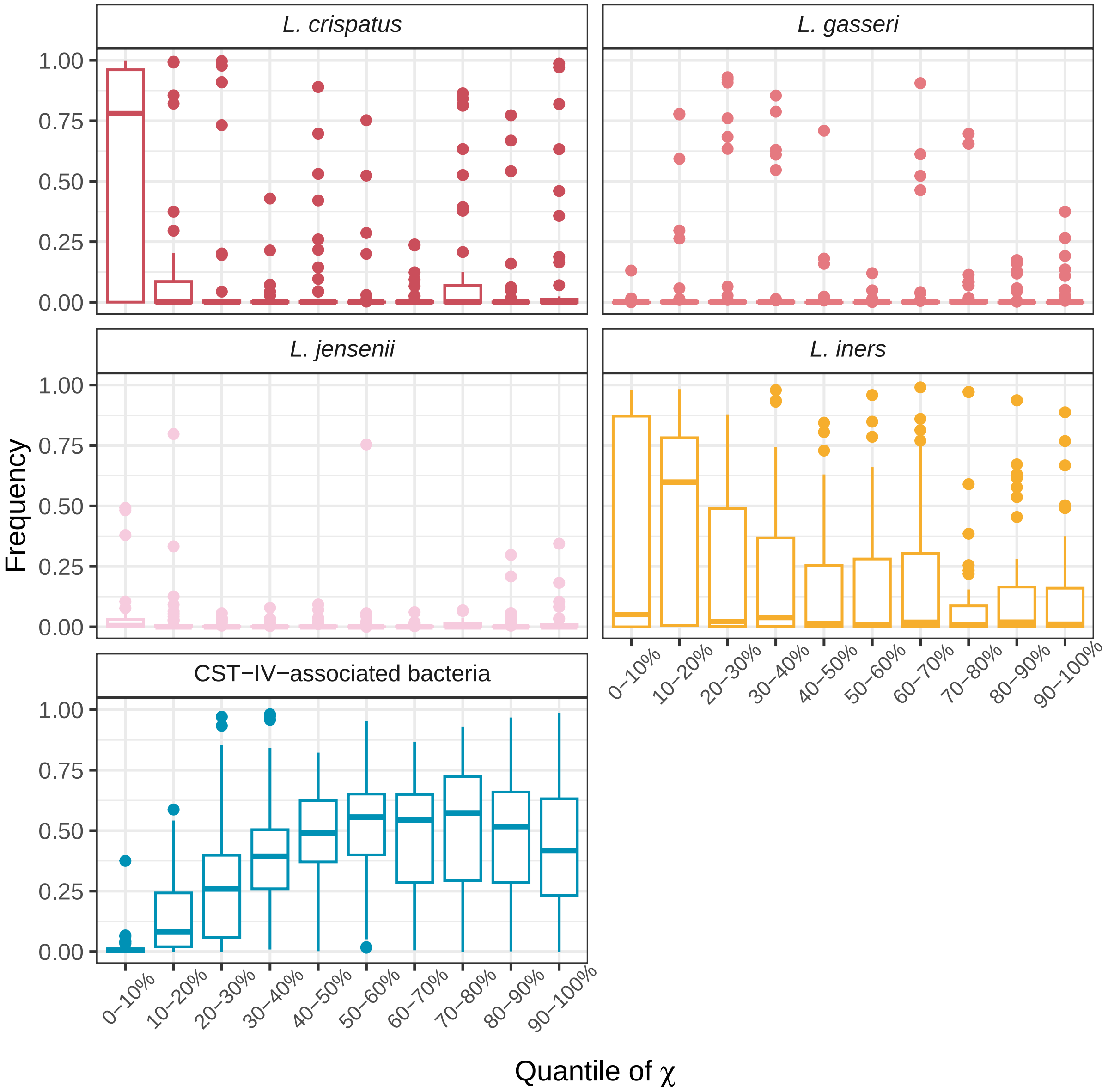

Figure S3: Frequency of vaginal microbiota species as a function of quantiles of empirically estimated χ (i.e., the proportion of supplied resource private to CST IV). Shown are *Lactobacillus* species associated with CST I (*L. crispatus*), II (*L. gasseri*), V (*L. jensenii*) and III (*L. iners*) and a sum of bacteria associated with CST IV according to VALENCIA. All the data and scripts used to generate the figure can be accessed at https://doi.org/10.57745/3GJF2Z.
